# Siglec-7 expression is reduced on a natural killer (NK) cell subset of obese humans

**DOI:** 10.1007/s12026-017-8942-y

**Published:** 2017-08-07

**Authors:** Philip Rosenstock, Rüdiger Horstkorte, Vinayaga Srinivasan Gnanapragassam, Jörg Harth, Heike Kielstein

**Affiliations:** 1 Institute for Physiological Chemistry, Martin Luther University Halle-Wittenberg, Hollystraße 1, 06114 Halle (Saale), Germany; 2 Department of Anatomy and Cell Biology, Martin Luther University Halle-Wittenberg, Grosse Steinstrasse 52 1, 06108 Halle (Saale), Germany; 3 Department of Transfusion Medicine, University Hospital Halle (Saale), Ernst-Grube-Straße 40, 06097 Halle (Saale), Germany

**Keywords:** CD56, FACS analysis, NK-cells, Obesity, Sialic acids, Siglec

## Abstract

Obesity leads to an altered adipocytokine production negatively effecting the function of natural killer cells (NK cells), which are important effector cells of the innate immune system. NK cells provide a defence against tumour cells or virus infected cells and have different activating and inhibitory surface receptors to distinguish between normal and transformed cells. One group of the inhibitory receptors are the sialic acid-binding immunoglobulin-like lectins (Siglecs). The aim of this study was to compare the expression of Siglecs-7, -9 and -10 on NK cells from normal weight and obese subjects. Therefore peripheral blood mononuclear cells (PBMC) were isolated from 10 normal weight (BMI < 25 kg/m^2^) and 11 obese (BMI > 30 kg/m^2^) blood donors and analysed by flow cytometry. Moreover, the amount of sialic acid on NK cell was determined using a fluorescent labelled lectin that binds terminal sialic acids. Percentages of immune cells were not altered between normal weight and obese individuals. CD56^bright^ NK cells from obese subjects had a reduced expression of Siglec-7 while the expression of Siglec-9 was not altered. The reduction of Siglec-7 expression on CD56^bright^ NK cells might be a marker for their dysfunction. Moreover, Siglecs-7, -9 and -10 are not expressed on the NK cell lines NK-92 and NKL. When comparing the two NK cell subpopulations CD56^bright^ and CD56^dim^, CD56^bright^ NK cells had a higher amount of sialic acids on their surface compared to CD56^dim^ NK cells regardless of body weight.

## Introduction

Obesity is one of the major health problems in high- and middle-income countries and has become a global epidemic during the last decades. In European countries, around 20% of the population is obese [1]. Obesity increases the risk of cardiovascular diseases and type II diabetes. However, it is also a major risk factor for several types of cancer, including liver, kidney or colon cancer [2]. Moreover, increased body weight is associated with a higher risk of postoperative wound infections [3] and a higher risk of infections in general [4]. All this indicates that obesity negatively acts on the immune system.

Natural killer cells (NK cells) are important effector cells of the innate immune system, capable of killing tumour cells and virus infected cells without antigen recognition [5]. In obese individuals, the leptin concentration in the blood is significantly increased as compared to normal weight subjects. Whereas, a short-term exposure to leptin has a stimulatory effect on NK cells, long-term exposure reduces the activity of NK cells and their IFN-γ production [6]. NK cells of obese individuals express less activation markers and show an altered NK cell phenotype [7]. These obesity-related alterations of NK cells are reversible, and the NK cell phenotype can be normalized by weight reduction [8].

In general, NK cells can be divided into two subpopulations based on their CD56 and CD16 expression. NK cells with a low CD56 expression (CD56^dim^) produce more CD16 and are more cytotoxic, whereas NK cells with a high CD56 expression (CD56^bright^) express less CD16 and produce immune-regulatory cytokines like IFN-γ, IL-10 or IL-13 [9]. CD56 negative NK cells are very rare and are mainly found during HIV infection [10].

The activity of NK cells is regulated by activating and inhibitory receptors. These include several receptor types, such as the natural cytotoxic receptors NKP46, NKP44, NKP30 or members of the killer immunoglobulin-like receptors (KIR) [11]. Another family of receptors represent the sialic acid-binding immunoglobulin-like lectins (Siglecs). There are 14 different Siglecs expressed by humans, mostly on cells of the immune system. Siglecs bind sialic acids via their C-type lectin domain from glycoconjugates (glycoproteins or glycolipids) on the same cell (cis) or on the surface of other cells (trans) [12].

NK cells express the Siglecs-7, -9 and -10 [13–15]. Since these Siglecs have an immunoreceptor tyrosine-based inhibition motif (ITIM) in their cytoplasmic domain, they could be considered as inhibitory receptors [16]. Siglecs-7, -9 and -10 belong to the so called CD33-related Siglecs, which are variable among different species and are all located in a gene cluster on chromosome 19 [17]. Since extracellular proteins are highly sialylated, Siglecs might be important to distinguish between self and non-self. A high amount of sialic acids on the surface protects tumour cells from NK cell lysis, and an enzymatic removal of sialic acids leads to a better killing of these cells by NK cells [18]. Therefore, overexpression of sialic acids provides an immune escape mechanism for tumour cells.

As mentioned above, the phenotype and functions of NK cells are altered in obesity. However, to date, nothing is known about the Siglecs expression on NK cells in obesity. Thus, the aims of the present study were (1) the investigation of the Siglec expression on human NK cells of normal weight and obese humans and on NK cell lines and (2) the quantification of sialic acids on NK cells.

## Materials and methods

### Cell lines

The human NK cell lines NK-92 and NKL were a kind gift from Dr. Roland Jacobs (Hannover Medical School). All cells were cultured in RPMI 1640 supplemented with 10% FCS (both from Biochrom AG, Berlin, Germany), 100 U*ml^−1^ penicillin and 100 mg* ml^−1^ streptomycin (both from Sigma–Aldrich, St. Louise, USA) 1 mM sodium pyruvate, 2 mM L-glutamine (both from Biochrom AG) in a 5% CO_2_ humified incubator (Thermo Fisher Scientific, Waltham, USA) at 37 °C. The medium for the NK cell lines was additionally supplemented with 200 U/ml human Interleukin-2 (IL-2) (Novartis Pharma GmbH, Zwickau, Germany).

### Study population

All study subjects were blood donors at the Department of Transfusion Medicine at the University Hospital in Halle (Saale). Each donor signed an agreement before using their blood samples. Subjects were divided into two groups based on their body mass index (BMI; kg/m^2^): obese with BMI > 30 kg/m^2^ (eight females, three males) and normal weight with BMI 18 kg/m^2−^25 kg/m^2^ (eight females, two males). All subjects suffered neither from any acute infection, immunosuppression or known malignant tumours in anamnesis.

### PBMC isolation

PBMC were isolated from the buffy coats of the blood donors using density gradient centrifugation. The buffy coats were diluted with PBS (Biochrom AG), and peripheral blood mononuclear cells (PBMC) were separated using biocoll separation solution (Biochrom AG). The interphase containing PBMC was collected and washed twice with PBS. Cell number was determined after trypan blue staining using and automated cell counter.

### Antibody staining

The cells were stained with the directly labelled mouse anti human antibodies CD3 conjugated with phycoerythrin (PE)-Cy7 (CD3-PE-Cy7) (clone SK7, 1:50, T cells), CD56 conjugated with allophycocyanin (CD56-APC) (clone NCAM16.2, 1:200, NK cells), CD16 conjugated with fluorescein isothiocyanate (CD16 FITC, 1:40) or conjugated with PE-CF594 (CD16-PE-CF594) (clone 3G8, 1:100), CD20 conjugated with allophycocyanin-HiliteV ®7-BD (CD20 APC-H7) (clone L27, 1:33, B cells) and CD14 conjugated with fluorescein isothiocyanate (CD14 FITC) (clone MφP9, 1:33, monocytes) (all BD Biosciences, San Diego, USA).

For the staining of the Siglecs, the directly labelled mouse anti human antibodies conjugated with PE Siglec-7-PE (clone 6–434, 1:50) (BioLegend, San Diego, USA), Siglec-9-PE (clone E10–286, 1:50) and Siglec-10-PE (clone 5G6, 1:25) (both BD Bioscience) and an isotype control were used (BD Bioscience).

Additionally, samples were stained with *Limax flavus* agglutinin (LFA) (EY Laboratories, San Mateo, USA) conjugated to Fluorescein (LFA-FITC) before staining with the antibodies to quantify the amount of sialic acids.

PBMC (1*10^6^ cells/100 μl) were incubated protected from light in a 96-well round bottom plate with the mentioned antibodies for 30 min on ice followed by two washing steps (PBS supplemented with 1% BSA and 0.1% sodium azide). Afterwards, a fixation with 1% paraformaldehyde in PBS for 10 min on ice was performed. Cells were washed, resuspended in measuring buffer (PBS supplemented with 0.1% BSA and 0.1% sodium azide) and analysed by flow cytometry.

### Flow cytometry

Flow cytometry was performed using a LSR Fortessa with BD FACSDiva Flow Cytometry Software Version 6.2 (BD Biosciences). Compensation was done with BD™ CompBeads Set Anti-Mouse Ig, κ (BD Biosciences). For gating the Siglec positive cells, a tube without Siglec antibodies (fluorescence minus one (FMO)) served as control. Furthermore, an isotype control was used to visualize possible unspecific binding of the antibodies to FC receptors.

Data was analysed using FACSDiva Flow Cytometry Software Version 6.2 and FlowJO 10 (FlowJo LLC, Ashland, USA).

### Statistical analysis

Data are presented as mean + SEM or as scatter plots including the median. Statistical analyses were performed using Student’s *t* test with the use of Graphpad Prism 5 Software (GraphPad Software, La Jolla, USA). *p*-values of less than 0.05 were considered significant.

## Results

The study population was composed of 21 subjects, which were divided into a normal weight group (BMI 18 kg/m^2^–25 kg/m^2^) and an obese group (BMI > 30 kg/m^2^). The study subjects were between 23 and 58 years old. No significant differences between the groups in age and height were found (Table 1), but the two groups significantly differed in their body weight (*p* < 0.0001) resulting in a significant difference in the BMI (*p* < 0.0001).

**Table 1:** Study population

	Normal weight (*n* = 11; 8 females, 3 males) mean ± SEM	Obese (*n* = 10; 8 females, 2 males) mean ± SEM	Significance
Age (years)	33.9 ± 2.5	41.4 ± 3.5	n.s.
Height (m)	1.73 ± 0.03	1.71 ± 0.02	n.s.
Weight (kg)	61.1 ± 1.7	114.1 ± 4.5	*p* < 0.0001
BMI	21.1 ± 0.4	38.9 ± 1.2	*p* < 0.0001

*SEM* standard error of the mean, *BMI* body mass index, *n.s*. not significant

### Immune cell populations are not altered in obese subjects

**Fig. 1:**
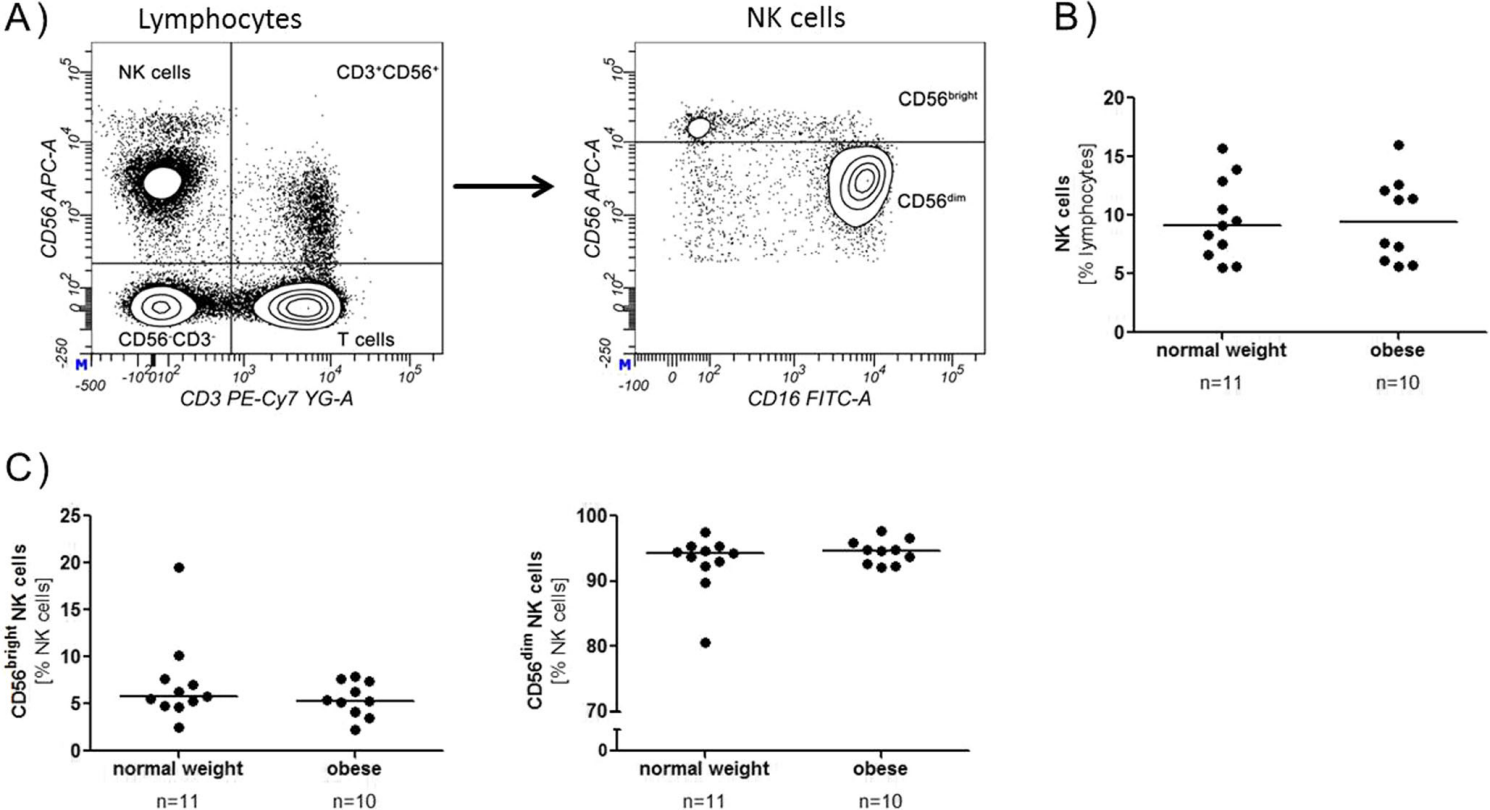
NK cells from normal weight and obese blood donors. PBMC were isolated from normal weight and obese blood donors, stained with different antibodies and analysed by flow cytometry. **a** NK cells were classified as CD56^+^CD3^−^ cells out of the lymphocytes and further divided into CD56^bright^ and CD56^dim^ NK cells based on their CD56 expression. The *contour plots* shown are from an obese donor. **b** Percentage of NK cells from normal weight and obese donors. **c** Percentage of CD56^bright^ and CD56^dim^ NK cells from normal weight and obese donors

NK cells were determined as CD3^−^CD56^+^ cells of the lymphocyte population. These cells can be further classified into two subpopulations, CD56^bright^ and CD56^dim^ NK cells, depending on their amount of CD56 expression. For a better separation of the populations, the CD16 expression was also analysed, because CD56^bright^ NK cells have a lower CD16 expression than CD56^dim^ NK cells (Fig. 1a). About 9% of all lymphocytes were NK cells in both groups with no significant differences between normal weight and obese subjects (Fig. 1b). Furthermore, no significant difference between normal weight and obese regarding the CD56^bright^ and CD56^dim^ NK cell numbers could be found (Fig. 1c). The percentage of T cells (CD3^+^), B cells (CD20^+^) and monocytes (CD14^+^) was also investigated and was not different between normal weight and obese subjects (data not shown).

### Human NK cell lines do not express Siglecs-7, -9 or -10

Human primary NK cells were analysed by flow cytometry for their expression of Siglecs-7, -9 and -10 and compared with two human NK cell lines, NK-92 and NKL. These two cell lines are commonly used as a model to study human NK cell function. Both, fluorescence minus one (FMO) and isotype controls indicated that no unspecific binding to Fc receptors occurred.

**Fig. 2:**
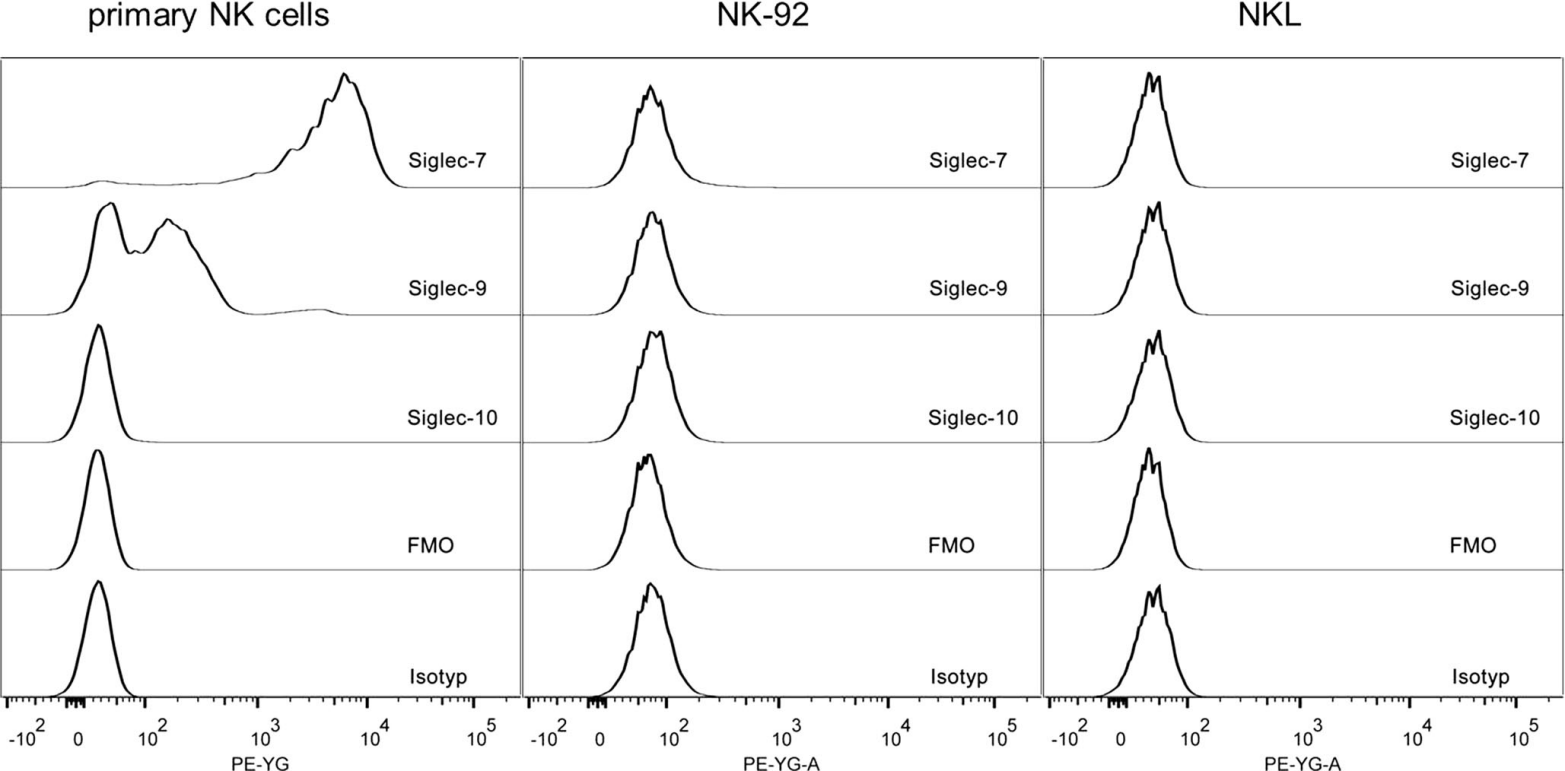
Siglec expression on NK cell lines NK-92 and NKL. The expression of Siglecs-7, -9 and -10 on human NK cells was analysed by flow cytometry and compared with the NK cell lines NK-92 and NKL. Primary NK cells were gated as shown in Fig. 1 and analysed for Siglec expression. A tube without Siglec antibodies (Fluorescence Minus One, FMO) and an isotype control were also used. Representative data from at least three independent experiments are shown

**Fig. 3:**
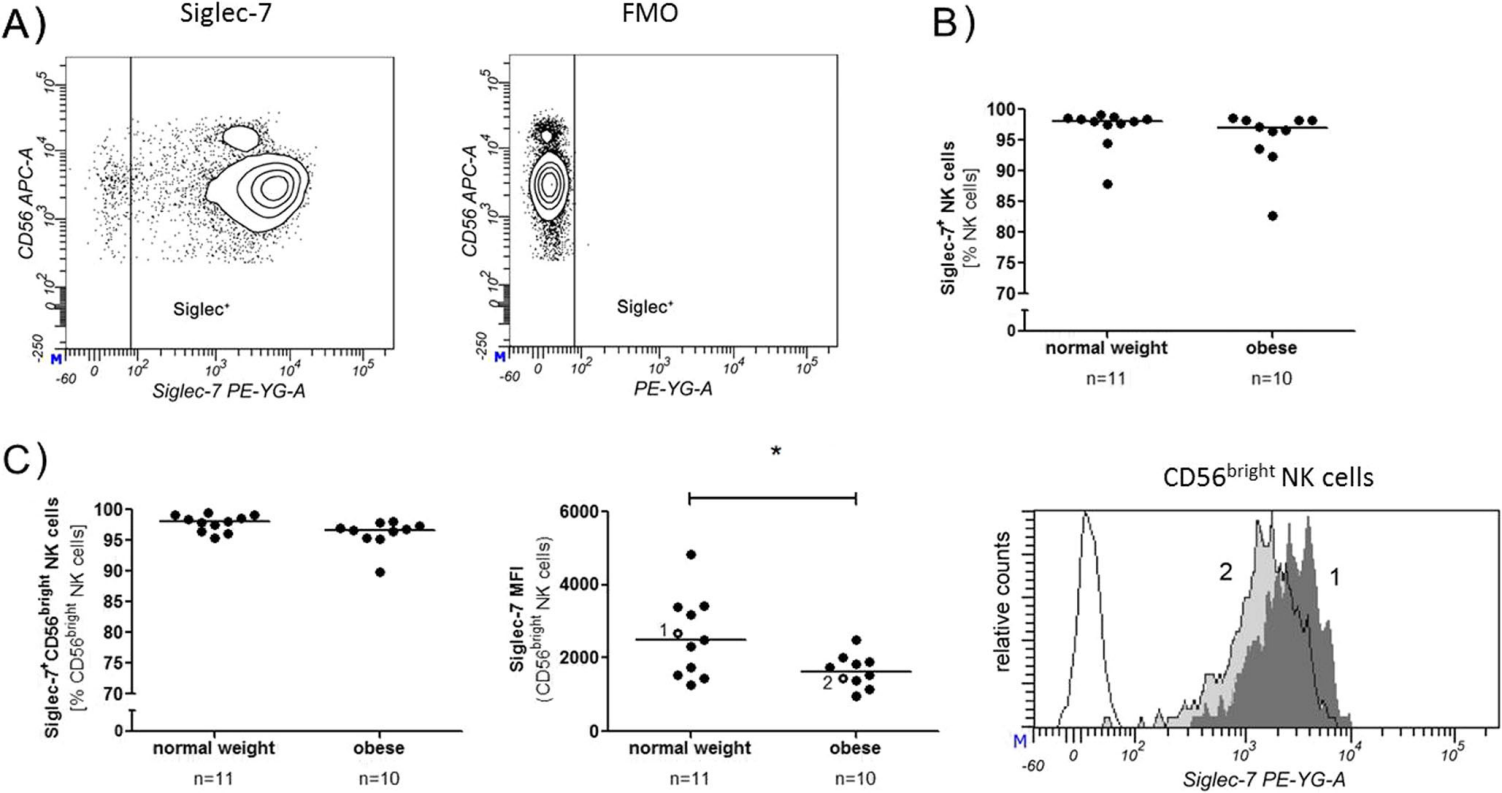
Siglec-7 expression. **a** NK cells were analysed for their Siglec-7 expression by flow cytometry. A tube without Siglec antibodies (Fluorescence Minus One, FMO) served as control to set the gates. The *contour plots* shown are from an obese donor. **b** Percentage of the Siglec-7^+^ NK cells from obese and normal weight donors. **c** Percentage of Siglec-7^+^ CD56^bright^ NK cells and median of the fluorescence intensity (MFI). Histogram of a representative normal weight (*1*, *dark grey*) and an obese donor (*2*, *light grey*) are shown together with the FMO control of the obese donor (*unfilled*). These donors were also marked in the scatter plot (*unfilled circles* with numbers *1* and *2*)

**Fig. 4:**
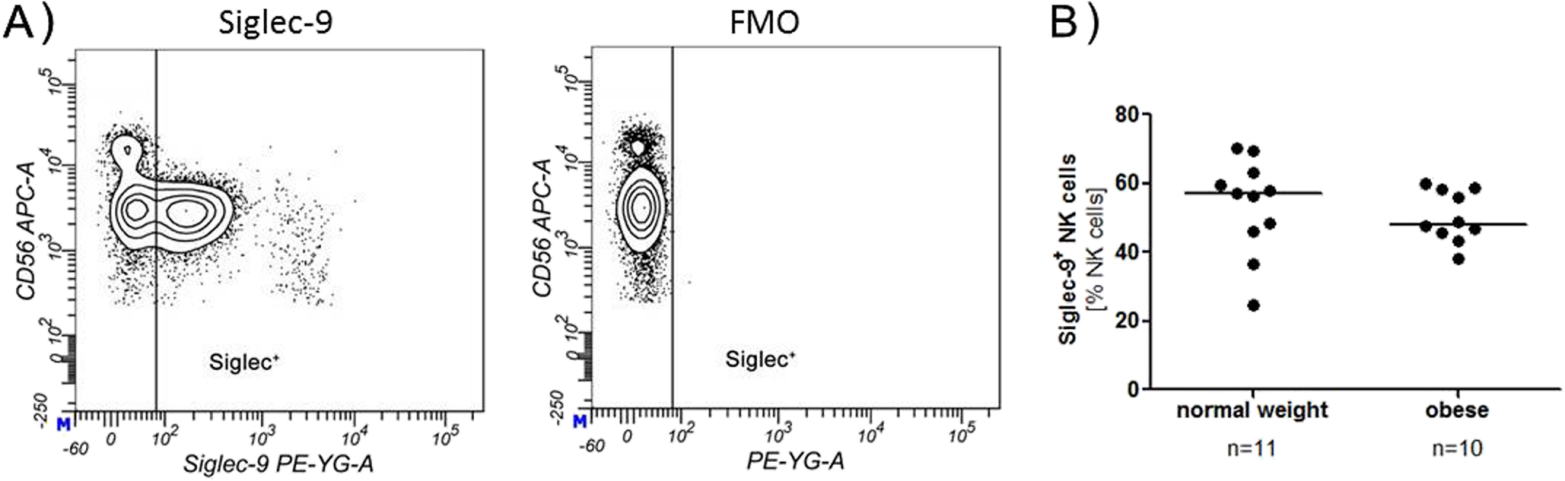
Siglec-9 expression. **a** NK cells were analysed for their Siglec-9 expression by flow cytometry. A tube without Siglec antibodies (Fluorescence Minus One, FMO) served as control to set the gates. The *contour plots* shown are from an obese donor. **b** Percentage of Siglec-7^+^ NK cells from obese and normal weight donors

Both cell lines showed no or only a weak expression (< 2%) of Siglecs-7, -9 and -10, when analysed by flow cytometry (Fig. 2). Comparing the results of these two cell lines with primary human NK cells, which express Siglec-7 by more than 95% and Siglec-9 by up to 75% (Fig. 3b and Fig. 4 b), Siglecs-7 and -9 were nearly absent on NK-92 and NKL. Siglec-10 however was hardly detectable both, on primary NK cells and on the cell lines (data not shown). Probably, its expression on NK cells might be restricted to tumour environment as described by Zhang et al. [14].

### Siglec-7 but not Siglec-9 expression is altered on CD56^bright^ NK cells in obesity

The Siglec-7 expression was analysed by flow cytometry, and percentage of Siglec-7 positive cells was measured together with the median fluorescence intensity (MFI) (Fig. 3a). For a general analysis of the Siglec-7 expression on NK cells, the data of all donors were combined (regardless the specific body weight), and CD56^bright^ and CD56^dim^ NK cells were compared. The percentage of Siglec-7 positive NK cells was not altered between the two subpopulations. However, CD56^bright^ NK cells had a significantly lower density of Siglec-7 compared with the CD56^dim^ NK cells, which could be revealed by a lower MFI. Since this was shown before by Shao et al. [19], we do not present these data here again.

The comparison of normal weight and obese donors showed no significant differences in the percentage of Siglec-7 expressing NK cells (Fig. 3b). The MFI and the percentage of Siglec-7 expressing CD56^dim^ NK cells were also not different between normal weight and obese donors. Even though the frequency of Siglec-7 positive cells was not altered for the CD56^bright^ NK cells, the analysis of the MFI showed a significantly lower Siglec-7 surface density on the CD56^bright^ NK cells of obese subjects compared with the same cells from normal weight subjects (Fig. 3c). Representative histograms from normal weight and obese donors are shown in Fig. 3c (right panel).

The Siglec-9 expression in general was significantly lower on the CD56^bright^ NK cells (2–15%) compared with the CD56^dim^ NK cells (40–75%), which could be revealed by both, percentage of Siglec-9 positive cells and MFI (data not shown) and was in accordance with data from Belisle et al. [20].

There were no significant differences in the Siglec-9 expression on NK cells between normal weight and obese subjects (Fig. 4b). Additionally, the Siglec-9 expression was determined for the two subpopulations separately, and no difference between normal weight and obese could be found either (data not shown).

T cells, B cells and monocytes were also analysed for their Siglec expression. No differences could be found between normal weight and obese subjects (data not shown). In general, Siglecs-7 and -9 were present on almost all monocytes. Siglec-10 could be found on the surface of some monocytes (~15%) and on B cells (~40%). T cells had no expression of Siglecs-7, -9 and -10.

### NK cell subpopulation shows different amounts of sialic acids

Sialic acids are the ligands for the Siglecs and are expressed either on other cells or on the NK cells itself. Therefore, the amount of sialic acid on NK cells itself was investigated. From 15 subjects (seven normal weight, eight obese), the relative amount of sialic acids on NK cells was determined using *Limax flavus* agglutinin (LFA) coupled with FITC, which binds to all terminal sialic acids, followed by the regular staining with the antibodies.

**Fig. 5:**
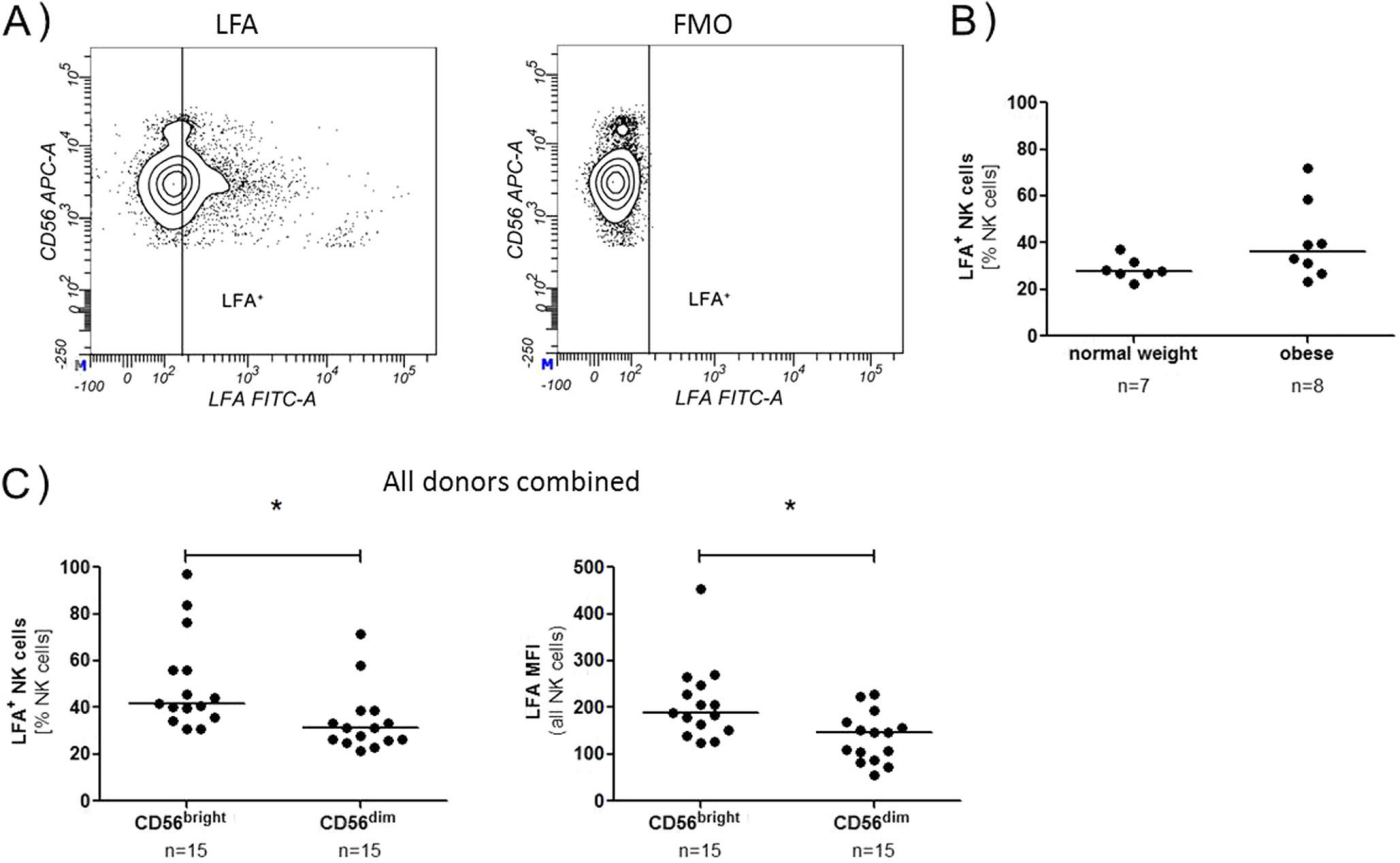
LFA staining. Prior to the antibody staining, cells were incubated with the lectin *Limax flavus* agglutinin (LFA) coupled with fluorescein. **a** NK cells were analysed for the binding of LFA by flow cytometry. Cells without the LFA incubation (Fluorescence Minus One, FMO) served as control to set the gates. The *contour plots* shown are from an obese donor. **b** Percentage of the LFA-binding NK cells from obese and normal weight donors. **c** The data from all donors were combined, and NK cells were grouped into CD56^bright^ and CD56^dim^ NK cells. Percentage of LFA-binding cells and MFI were measured

NK cells were again gated as CD3^−^CD56^+^ cells, and the percentage of LFA binding cells and MFI were measured (Fig. 5a). There were no significant differences between normal weight and obese donors, even though NK cells from the obese group showed an enhanced LFA binding as compared to the cells from the normal weight group (Fig. 5b).

For a general comparison between CD56^bright^ and CD56^dim^ NK cells, data from all 15 donors were combined. The CD56^bright^ NK cells had a significantly higher MFI and a higher percentage of LFA-binding NK cells than CD56^dim^ NK cells, indicating that these cells have more sialic acids on their surface (Fig. 5c). Differences in the phenotype of CD56^bright^ and CD56^dim^ NK cells are summarized in Table 2.

**Table 2:** Phenotype of NK cell subpopulations

	CD16	Siglec-7	Siglec-9	Sialic acids
CD56^bright^	No or only weak expression	> 90% positive but lower expression	2–15% positive	High expression of sialic acids
CD56^dim^	High expression	> 90% positive	40–75% positive	Low expression of sialic acids

## Discussion

It has been shown previously that the NK cell function and phenotype are altered in obesity. The present study shows no differences in the percentage of NK cells, as well as the percentage of T cells, B cells and monocytes between normal weight and obese subjects. These data are in accordance with previously published data, showing stable NK cell numbers with significantly decreased functional parameters in obese humans [7].

The NK cell lines NK-92 and NKL are common cell lines used to study human NK cells in vitro. Here, we show that these cells do not express Siglecs-7 and -9, which are normally expressed by primary NK cells. Therefore, these cell lines are not suitable for analysing the function of Siglecs on NK cells without a further manipulation such as transfection to express Siglecs-7 and -9. Since the focus of our study was the evaluation of effects of an obese metabolic environment on the Siglec expression of NK cells, we decided to analyse primary NK cells of normal weight and obese healthy humans.

The majority of human NK cells in healthy subjects express Siglec-7. However, in the course of several infections, such as hepatitis C virus (HCV), the number of Siglec-7 expressing NK cells decreases. Reduction of Siglec-7 expression is associated with a dysfunctional NK cell phenotype, reduced degranulation and cytokine secretion. Furthermore, the number of Siglec-7^neg^ NK cells is elevated, which correlates with markers of liver injury and fibrosis in patients with chronic HCV [21]. Siglec-7 expression on NK cells is also decreased in patients with HIV infection [22]. In general, Siglec-7 expressing NK cells are more functional than Siglec-7^neg^ NK cells and display a more active phenotype with a higher expression of several activation markers and a higher cytokine production [19]. In this study, we could show that Siglec-7 is downregulated on CD56^bright^ NK cells in obese humans. Apart from the highly cytotoxic CD56^dim^ NK cells, CD56^bright^ NK cells represent an NK cells subset that is only weakly cytotoxic. However, these cell type is important for the production of cytokines such as IFN-γ, and thereby activate other immune cells like dendritic cells or monocytes [23]. Several studies have shown various impairments of NK cell functions, e.g. cytokine production, granzyme expression and migration in obese individuals [7, 24]. Even though the total number of Siglec-7 expressing NK cells is not altered, it can be hypothesised that a lower expression of Siglec-7 is associated with an impaired NK cell function. With the results of the present study, another altered functional parameter of NK cells in obese subjects is added. Siglec-7 was recently shown to have a striking preference for internally branched α-2,6-linked di-sialic gangliosides such as DSGb5 and disialosyl Lc_4_ (DSLc4), and α-2,8-linked gangliosides such as GD2, GD3, or GT1b [25, 26]. Previous studies have shown that interactions between Siglec-7 and GD3 modulate the cytotoxic activity of NK cells and that Siglec-7 is constitutively masked on NK cells. Unmasking of Siglec-7 by sialidase-treatment can lead to an interaction with GD3 on potential target cells and interferes with NK cell cytotoxic activities [27]. Unlike Siglec-7, the expression of Siglec-9 was not altered between normal weight and obese subjects. Siglec-9 is mainly expressed by CD56^dim^ NK cells and only weakly expressed on CD56^bright^ NK cells [20].

Apart from the Siglec expression, the sialylation of NK cells was also investigated in this study. Sialic acids represent the terminal monosaccharides of most glycoproteins and glycolipids and can masked the carbohydrate recognition domains of Siglecs via cis-interactions [28]. In this study, we could show that sialylation of CD56^bright^ NK cells is higher compared with sialylation of CD56^dim^ NK cells. High concentrations of sialic acids on the surface provide more masking ligands for the Siglecs. Together with the fact, that CD56^bright^ cells have a lower expression of both, Siglecs-7 and -9 it could be assumed, that the masking effect is very strong on this cell type compared with CD56^dim^ NK cells. As CD56^bright^ and CD56^dim^ NK cells differ in their receptor expression, the differences in sialic acid levels can be due to the expression of more sialylated proteins or might be the reason of differences in the expression of sialyltransferases. Nevertheless, the higher concentration of sialic acids on the surface of CD56^bright^ NK cells is another different phenotype marker which distinguishes CD56^bright^ and CD56^dim^ NK cells and has not been described so far. More investigations are necessary to analyse the reason for the higher level of sialic acids on the surface of CD56^bright^ NK cells.

In summary, this study showed that obesity leads to a reduction of Siglec-7 expression on CD56^bright^ NK cells which might be a marker of NK cells dysfunction. Apart from differences in the Siglecs-7 and -9 expressions, CD56^bright^ NK cells have a higher amount of sialic acids on their surface compared with CD56^dim^ NK cells regardless of the individual body weight.
